# The Effect of Student Pharmacists as Peer Coaches on Implementation of Community Pharmacy Services

**DOI:** 10.5888/pcd23.260472

**Published:** 2026-11-05

**Authors:** Abigail R. Morgan, Kaileigh D. Yen, Jasmine Strayhorn, Stefanie P. Ferreri

**Affiliations:** 1 Eshelman School of Pharmacy, University of North Carolina at Chapel Hill, Chapel Hill, North Carolina; 2 Clinical Engagement and Professional Advancement, Blue Cross and Blue Shield North Carolina, Durham, North Carolina

## Abstract

**Purpose and Objectives:**

Pharmacist-led chronic disease management services can lead to improvements in health outcomes, yet barriers exist to the implementation of these services in community pharmacies. This study aimed to assess the effect of student pharmacists serving as peer coaches during implementation of a disease management program in community pharmacies.

**Intervention Approach:**

In summer 2025, student pharmacists served as peer coaches to facilitate implementation of a program designed to manage type 2 diabetes and hypertension among pharmacy patients into the workflow of participating pharmacies.

**Evaluation Methods:**

A 35-item questionnaire was emailed to participating pharmacy teams before and 30 days after the coaching intervention. Survey questions assessed pharmacy characteristics, perceived success, readiness for implementating change, and attitudes toward the More Than a Script program. Program engagement data (number of patient encounters and number of completed patient visits) were collected from May to September 2025. We used the number of completed patient visits to calculate revenue generated during the coaching intervention. We used descriptive and comparative statistics for analysis.

**Results:**

Of 29 pharmacy teams participating in the coaching intervention, 18 (62%) responded to the survey. Pharmacy personnel indicated improvements in perceived success, readiness for change, and attitudes toward the program. The number of completed patient visits per pharmacy by the end of the coaching intervention was higher among coached pharmacy teams than among uncoached pharmacy teams with the exclusion of an outlier in the uncoached group (28 vs 13, *P* = .001). The average percentage of total patient encounters that resulted in a completed patient visit increased more in the coached group (from 32% to 56%) than in the uncoached group regardless of the outlier (from 22% to 25% with the outlier, 21% to 24% without the outlier; *P* = .02). The average revenue per pharmacy was $1,005 for coached pharmacies and $274 for uncoached pharmacies after excluding an outlier in the uncoached group (*P* = .001).

**Implications for Public Health:**

The accessibility offered by community pharmacies provides a path to connect patients to chronic disease management, but improved outcomes cannot occur without effective implementation. Student pharmacists serving as peer coaches can help community pharmacies with implementation of services to improve population health outcomes.

**Summary**

**What is already known on this topic?**

Chronic disease is a leading cause of illness and death in the US. Pharmacist-led services have been shown to improve outcomes for patients with diabetes and hypertension.

**What is added by this report?**

Student pharmacists acting as peer coaches can serve as a successful implementation strategy for integrating pharmacist-led clinical services into community pharmacies.

**What are the implications for public health practice?**

Pharmacists are among the most accessible health care providers, making them well-positioned to offer chronic disease management services. Integrating these services into community pharmacies can improve population health outcomes.

## Introduction

The shortage of primary care providers in the US is estimated to be 20,000 to 40,000 providers by 2036 (1). With the prevalence of chronic diseases also rising, creative solutions for managing chronic diseases are needed (2). Type 2 diabetes (henceforth, diabetes) and hypertension are 2 of the most common chronic diseases in the US, and complications such as heart disease, stroke, and kidney disease are among the top 10 causes of death in the country (3,4). In addition, diabetes and hypertension cost the US more than $500 billion each year in health care spending and lost economic productivity (5,6). Although health insurance payors can often identify high-cost patients, they may lack a standardized and reliable approach for connecting patients to health care providers in areas with shortages of primary care providers. This lack of accessibility reduces opportunities to improve patient outcomes and curb health care expenditures.

Patients visit pharmacists twice as often as they visit other health care professionals (7). This accessibility positions pharmacists to support patients and payors in value-based arrangements (8). Pharmacists’ role as medication experts allows them to support patients in managing chronic conditions, such as diabetes and hypertension, for which lifestyle and medication interventions can alter patient outcomes. Pharmacist-delivered services have been proven to significantly reduce glycated hemoglobin A_1c _(HbA_1c_) in patients with diabetes and improve blood pressure outcomes in patients with hypertension (9,10).

An opportunity for community pharmacists to engage in chronic disease management services became available when a payor in North Carolina, Blue Cross and Blue Shield of North Carolina (Blue Cross NC), identified the need to improve diabetes and hypertension outcomes for its beneficiaries. The More Than a Script (MTS) program, a value-based care initiative, was developed by Blue Cross NC in collaboration with community pharmacies and a health care technology vendor. MTS operationalizes pharmacist-led interventions to deliver comprehensive services for patients with diabetes and hypertension, including structured education, pharmacotherapeutic optimization, and interprofessional care coordination. Blue Cross NC partnered with a pharmacy advisory council composed of representatives from the state pharmacy organization, community pharmacy networks, and academic institutions to design the MTS program. MTS primarily serves fully insured Blue Cross NC commercial members and Medicare beneficiaries diagnosed with diabetes and/or hypertension. MTS launched in June 2024 with a phased rollout, followed by a relaunch in March 2025. As part of the relaunch, an internship program with the University of North Carolina at Chapel Hill (UNC) Eshelman School of Pharmacy introduced student pharmacists as peer coaches to assist community pharmacy teams with implementation of MTS.

Peer coaches are colleagues who educate others without having formal authority over those they are teaching (11). Peer coaching can be an effective method for improving outcomes across various professions (11), including community pharmacies, where peer coaching has been shown to promote the adoption of proactive workflow practices and facilitate the implementation of expanded clinical services (12,13). Additionally, peer coaches can help identify site champions at pharmacies. Site champions can support and drive implementation by promoting positive behavior change and overcoming barriers to implementation (14,15).

## Purpose and Objectives

The purpose of this study was to assess the effect of student pharmacists serving as peer coaches for implementation of MTS at community pharmacies in North Carolina.

## Intervention Approach

In our intervention, 4 rising third-year pharmacy students served as peer coaches through a paid summer internship (May through August 2025) with the Eshelman School of Pharmacy. Prior to engaging with pharmacies, interns completed an orientation in early May 2025, followed by 3 weeks on-site at a pilot pharmacy. Students were educated on the Flip the Pharmacy (FtP) initiative before engaging with pharmacy personnel. FtP is a practice transformation initiative that aims to create longitudinal, patient-level care processes and business models to facilitate the delivery of patient care services (16). FtP endorses 6 domains of practice transformation activities that create a workflow that is more predictable and holistic than a traditional pharmacy workflow, allowing for more opportunities to integrate health care services for patients with chronic diseases. The 6 domains are leveraging the appointment-based model, improving patient follow-up and monitoring, developing new roles for nonpharmacist support staff, optimizing the use of technology, establishing working relationships with other care team members, and developing the business model and expressing value (16). Excelling in these domains creates a proactive approach that can promote sustainability, encourage interprofessional collaboration, and streamline workflow (17). Prior research demonstrated that the FtP initiative was a helpful strategy for implementing and improving community pharmacy-based patient care (18). Using the FtP approach can result in fewer barriers to implementation when pharmacies engage in payor-sponsored clinical services, allowing for the adoption of revenue streams beyond traditional fee-for-service dispensing (19,20). Due to its documented success in community pharmacies, we used the FtP domains and model in our student peer coaching model for implementation of MTS.

Student peer coaches were instructed to assess baseline pharmacy performance using the FtP domains and identify areas for improvement during the coaching process. Although FtP provides a blueprint for pharmacy personnel to improve workflow, it does not assess a pharmacy team’s readiness to implement these workflow changes. Having peer coaches assess readiness allowed for implementation efforts to be tailored to each pharmacy’s needs.

From June through mid-August 2025, student pharmacist interns engaged with pharmacy teams both in person and virtually. The length of time spent coaching each pharmacy team and the techniques used when coaching were tailored to baseline pharmacy performance. Examples of student pharmacist interventions included optimizing proactive medication refills, identifying patients engaged in other expanded services (eg, remote patient monitoring, adherence packaging, medication synchronization), educating pharmacy personnel on navigating technology vendors, creating materials to advertise the MTS program to patients, securing access to electronic health records, educating pharmacy personnel on MTS program contents, and involving all pharmacy personnel in MTS program activities. Student peer coaches also identified site champions to improve implementation efforts. Site champions served as the designated point of contact for other pharmacy personnel to approach with questions or concerns about the MTS program and took the lead on sustained program activity after completion of the intervention. Site champions were not limited to a specific job title; identification of site champions was based on the resources and needs of each pharmacy team. Student peer coaches worked closely with site champions to promote implementation and sustainability of the service after coaching was completed.

Pharmacies with the largest number of patient engagement opportunities and locations closest to the Eshelman School of Pharmacy were prioritized for initial coaching support. These pharmacies were identified by Blue Cross NC and contacted by the faculty members overseeing the student peer coaches. Faculty members assessed site interest and enrolled pharmacies in the coaching program. Pharmacy teams could request that student peer coaching take place on site, virtually, or a hybrid of the two. For on-site assistance, student peer coaches worked closely with the pharmacy team to determine the amount of time needed on site. For some of the on-site locations, student peer coaches followed up virtually with the pharmacy team after site visits occurred, creating the hybrid model. Once pharmacies from this prioritized list were offered the opportunity to enroll in the coaching program, all pharmacies in the MTS program were offered the opportunity to schedule a virtual coaching session, regardless of geographic location or number of patients.

## Evaluation Methods

### Study design and setting

This evaluation was based on a web-based pre- and postintervention survey conducted among community pharmacies that were enrolled in the MTS program and engaged in the student peer coaching intervention. The study was determined to be exempt by the institutional review board at UNC (no. 25–1795).

### Survey participants and procedures

Each pharmacy that was engaged in the on-site, virtual, or hybrid student peer coaching intervention was invited to participate in a 35-item survey developed and distributed using Qualtrics software (Qualtrics) before the intervention (June 19, 2025) and after the intervention (August 5, 2025). Surveys were available for 8 weeks. Instructions requested that the pharmacy team member most familiar with the MTS program complete the survey. The job title of the respondents was not recorded. The surveys collected the following data on pharmacy characteristics: student peer coaching type (on-site, virtual, or hybrid), patient scheduling method used (MTS technology vendor, free third-party vendor, paid third-party vendor, video conferencing, or paper), access to electronic health records (yes or no), number of pharmacy personnel involved in the MTS program, and hours spent per week on the MTS program by pharmacy personnel. Student peer coaches individually tracked the number of hours they spent coaching each pharmacy team. The first 2 pharmacy teams to participate in the intervention served as pilot sites and were excluded from participating in the surveys.

The surveys used Likert-like scales to assess perceived success, readiness to implement change, and attitudes toward the MTS program. Perceived success questions addressed proficiency in 5 of the 6 FtP domains: workflow integration, team engagement, patient engagement, software optimization, and provider relationships. Readiness questions assessed respondents’ willingness to change based upon a modified Organizational Readiness for Implementing Change framework (21). The survey also assessed attitudes of survey respondents about knowledge of the MTS program, effectiveness of MTS program integration into workflow, overall satisfaction with the MTS program, and likelihood of recommending peer coaching. Components of knowledge of the MTS program included confidence in completing patient visits, navigating the technology vendor platform, locating relevant resources, and familiarity with the goals of the program. These data provided context for pharmacy behavior and changes in perspective throughout the coaching intervention, with survey questions emphasizing changes resulting from student peer coaching.

### Patient encounters

The MTS technology vendor collected data on patient encounters for all pharmacies involved in the MTS program, regardless of whether the pharmacy participated in the student peer coaching intervention. These data were collected from before and after the student peer coaching intervention. The preintervention data included all patient encounters from the date the pharmacy first enrolled in the MTS program until May 5, 2025, and postintervention data included all patient encounters from the date the pharmacy first enrolled in the MTS program until September 1, 2025. We summed the number of patient encounters across all pharmacies and calculated the average number of patient encounters per pharmacy. We found an outlier in the uncoached group: this pharmacy had a pharmacist dedicated solely to the MTS program, so the number of patient encounters at this pharmacy substantially exceeded the overall group average. We summarized patient encounter data both with and without this outlier.

Patient encounter data included both completed and uncompleted patient visits. Completed patient visits were defined as encounters in which pharmacy personnel successfully enrolled the patient into the MTS program, provided educational services, documented visit details, and submitted information for reimbursement to Blue Cross NC. Uncompleted patient visits were defined as encounters in which pharmacy personnel did not enroll the patient in the MTS program (eg, patient did not follow up after outreach, patient declined participation, or patient switched insurance providers). We calculated the percentage of patient encounters that resulted in a completed patient visit for each pharmacy. We used the number of completed patient visits to calculate revenue generated by each pharmacy during the coaching intervention. These outcomes provide insight into program engagement between coached and uncoached pharmacy teams.

### Data collection and analysis

Preintervention surveys were initially sent on June 19, 2025. Postintervention surveys were initially sent on August 5, 2025. We used a modified Dillman approach (22) for survey reminders. Survey data collection closed September 30, 2025. Although any pharmacy team receiving the intervention was classified as a coached pharmacy, only those pharmacy teams that completed both the pre- and postintervention surveys were included in the analysis of survey-based outcomes. We used descriptive statistics to analyze pharmacy characteristics, student-reported hours, perceived success by FtP domain, readiness for implementing change (12 questions analyzed as a single composite measure), and attitudes toward the MTS program. Of the attitudes examined, we analyzed knowledge of program components as a single composite measure (4 questions) and assessed workflow integration, overall satisfaction, and likelihood to recommend coaching individually.

We included all coached pharmacy teams, except the 2 pilot locations, in the analysis of encounter data regardless of whether pharmacies responded to the survey. We analyzed data on program encounters and revenue generated during the coaching intervention using comparative statistics for both coached and uncoached pharmacy teams. Because of the unequal sizes of the 2 pharmacy groups, we used the Welch 2-tailed *t* test to compare patient encounter data between the coached and uncoached teams. We calculated revenue by using the number of completed patient visits and a reimbursement rate of $50 per patient visit. Significance for all analyses was set at *P* < .05.

## Results

### Survey responses

During the study period, 81 pharmacy sites were enrolled in the MTS program; 29 teams completed the student peer coaching intervention (coached), and 52 teams did not complete the intervention (uncoached). Among the 29 pharmacy teams that completed the intervention, 18 completed both the pre- and postintervention surveys (response rate, 62%). Of the 18 teams, 8 were coached on-site, 8 virtually, and 2 using the hybrid model. The number of pharmacy personnel who engaged in the MTS program increased from 29 to 55 across all 18 pharmacies (Table 1). The number of hours spent by pharmacy personnel on the MTS program per week decreased from 91 to 85 across all 18 pharmacies. Student peer coaches spent an average of 52.9 hours per pharmacy at on-site locations, 2.8 hours per pharmacy using the virtual model, and 70.8 hours per pharmacy using the hybrid model. For the 2 pharmacies using the hybrid model, 96% (136 of 141.5) of the coaching hours were spent on site.

**Table 1:** Characteristics of Pharmacies (N = 18) Before (Preintervention) and After (Postintervention) Peer Coaching by Pharmacy Students, North Carolina, 2025

Characteristic	Preintervention^a^	Postintervention^b^
**Patient scheduling method used by pharmacy**		
More Than a Script technology vendor	12	12
Free third-party vendor	4	6
Paid third-party vendor	4	6
Video conferencing	2	1
Paper	4	3
**Electronic health record access**		
Yes	10	13
No	8	5
**More Than a Script program involvement**		
No. of pharmacy personnel	29	55
No. of hours spent per week by pharmacy personnel	91	85

a Data collected from surveys sent June 19, 2025.

b Data collected from surveys sent August 5, 2025.

The number of survey respondents (n = 18) rating themselves “above average” or “excellent” increased from the start to the end of the coaching interventions in each of the 5 examined FtP domains (Figure 1). We observed the greatest increases in software optimization (from 3 to 11 respondents), followed by patient engagement (from 4 to 9 respondents), team engagement (from 3 to 8 respondents), workflow integration (from 3 to 7 respondents), and provider relationships (from 6 to 9 respondents).

**Figure 1:**
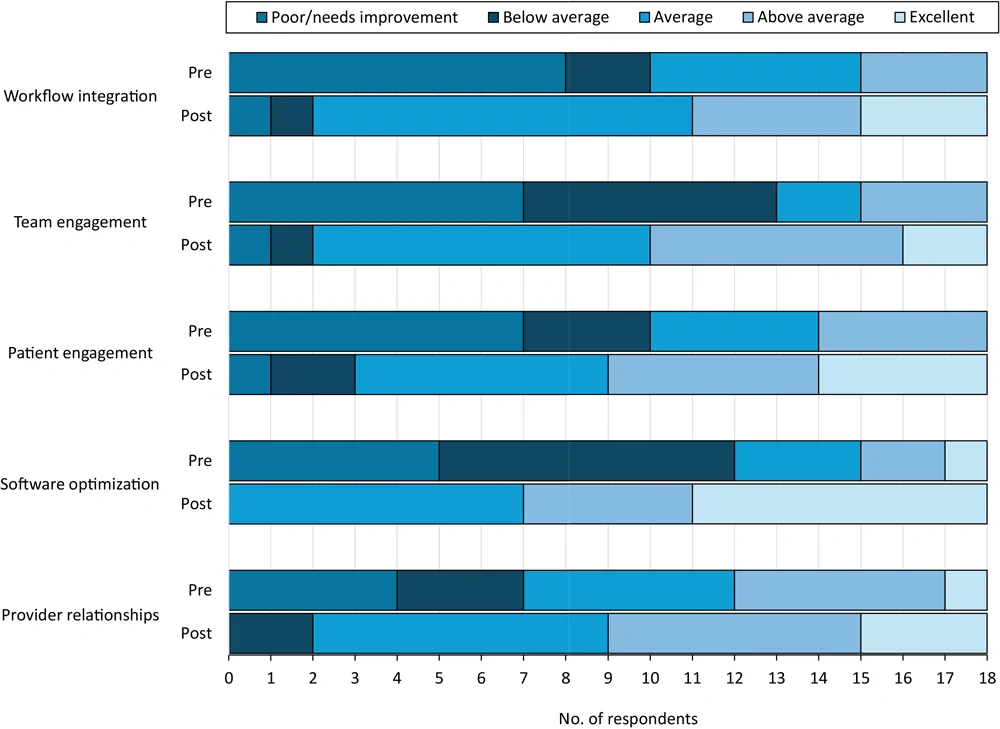
Perceived success in each of 5 Flip the Pharmacy domains before and after the student peer coaching intervention, as rated by 18 survey respondents employed at pharmacies enrolled in the More Than a Script program, North Carolina, 2025.

**Table:** 

Domain	Provider relationships		Software optimization		Patient engagement		Team engagement		Workflow integration	
	Post	Pre	Post	Pre	Post	Pre	Post	Pre	Post	Pre
Poor/needs improvement	0	4	0	5	1	7	1	7	1	8
Below average	2	3	0	7	2	3	1	6	1	2
Average	7	5	7	3	6	4	8	2	9	5
Above average	6	5	4	2	5	4	6	3	4	3
Excellent	3	1	7	1	4	0	2	0	3	0

The percentage of respondents selecting “somewhat agree” or “strongly agree” to the 12 questions about readiness for change increased from 55% (117 of 216) to 66% (142 of 216) (Figure 2). The percentage of respondents selecting “somewhat agree” or “strongly agree” to the 4 MTS program knowledge questions increased from 67% (48 of 72) to 100% (72 of 72). Those describing MTS activities as adequately integrated into pharmacy workflow increased from 4 to 10 respondents. Those who were satisfied overall with the MTS program increased from 8 to 14. When asked how likely they were to recommend a student pharmacist coach to their peers, 6 of 8 participants receiving virtual support, 7 of 8 receiving on-site support, and 2 of 2 receiving hybrid support reported they would be extremely likely to do so.

**Figure 2:**
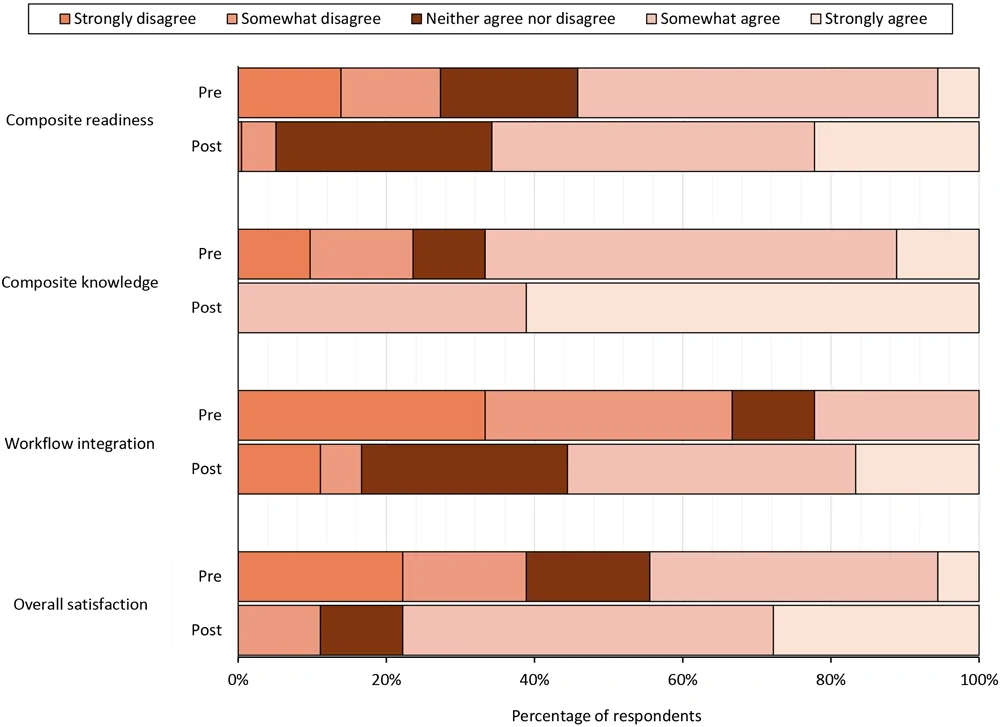
Readiness for and attitudes toward the More Than a Script program before and after the student peer coaching intervention, as rated by 18 survey respondents employed at pharmacies enrolled in the More Than a Script program, North Carolina, 2025. “Knowledge” is a composite measure of 4 questions that assessed knowledge of More Than a Script program components among pharmacy teams. “Readiness” is a composite measure of 12 questions that assessed readiness for change among pharmacy teams. The stronger the agreement, the greater the overall satisfaction, workflow integration, knowledge, and readiness for change.

**Table:** 

Response	Overall satisfaction, %		Workflow integration, %		Knowledge, %		Readiness, %	
	Post	Pre	Post	Pre	Post	Pre	Post	Pre
Strongly disagree	0	22.2	11.1	33.3	0.0	9.7	0.5	13.9
Somewhat disagree	11.1	16.7	5.6	33.3	0.0	13.9	4.6	13.4
Neither agree nor disagree	11.1	16.7	27.8	11.1	0.0	9.7	29.2	18.5
Somewhat agree	50.0	38.9	38.9	22.2	38.9	55.6	43.5	48.6
Strongly agree	27.8	5.6	16.7	0.0	61.1	11.1	22.2	5.6

### Patient encounters

Coached pharmacy teams had a higher number of completed patient visits per pharmacy than uncoached pharmacy teams by the end of the coaching intervention, excluding the outlier (28 vs 13, *P* = .001) (Table 2). The average percentage of total patient encounters that resulted in a completed visit increased more in the coached group (32% to 56%) than in the uncoached group regardless of the outlier (22% to 25% with, 21% to 24% without, *P* = .02). An average revenue of $1,005 per coached pharmacy and $1,049 per uncoached pharmacy was generated from May 5 to September 1 when including the outlier (*P* = .96). When the outlier was excluded, the average revenue per uncoached pharmacy dropped to $274 (*P* = .001).

**Table 2:** Patient Encounters Preintervention^a^ and Postintervention^b^ for Coached and Uncoached Pharmacies in an Intervention That Placed Student Pharmacists in Community Pharmacies as Peer Coaches, North Carolina, 2025

Outlier vs no outlier	Coached pharmacies (n = 29)		Uncoached pharmacies (n = 52)		*P* value^c^
	Preintervention	Postintervention	Preintervention	Postintervention	
**With outlier^d^**					
Patient encounters					
Total no.	987	3,015	1,158	2,753	.20
Mean (SD) per pharmacy	34.0 (72.4)	104.0 (150.6)	22.2 (96.3)	52.9 (182.3)	
Completed patient visits					
Total no.	195	818	428	1,519	.96
Mean (SD) per pharmacy	6.7 (10.1)	28.2 (24.5)	8.2 (22.2)	29.2 (122.3)	
**Without outlier^d^**					
Patient encounters					
Total no.	987	3,015	1,113	1,854	.02
Mean (SD) per pharmacy	34.0 (72.4)	104.0 (150.6)	21.8 (96.3)	36.4 (182.3)	
Completed patient visits					
Total no.	195	818	384	663	.001
Mean (SD) per pharmacy	6.7 (10.1)	28.2 (24.5)	7.5 (21.9)	13.0 (36.4)	

a All-time data from enrollment in the More Than a Script program until May 5, 2025.

b All-time data from enrollment in the More Than a Script program until September 1, 2025.

c Welch 2-tailed *t* test used to determine *P* value.

d A pharmacy in the uncoached group had a pharmacist dedicated solely to the More Than a Script program, so the number of patient encounters at this pharmacy was substantially higher than in the overall group average, and the pharmacy was considered an outlier. The average percentage of total patient encounters that resulted in a completed patient visit increased more in the coached group than the uncoached group (*P* = .02), regardless of outlier inclusion. The number of uncoached pharmacies without the outlier was 51.

## Implications for Public Health

Our study showed that peer coaching by student pharmacists improved perceived success in FtP domains, readiness for change, attitudes toward the MTS program, and patient engagement. When a student pharmacist who is not a pharmacy employee assumes the student peer coaching role, a mutually beneficial arrangement that prioritizes an outside perspective, accountability, and leadership development is created (23). Because students who participate in pharmacy practice experiences usually spend a limited amount of time with a pharmacy team, they can provide an outsider’s insight — insight that pharmacy employees may not be able to offer. Pharmacy personnel may be more likely to prefer a traditional workflow over new approaches due to repeated exposure to a routine and the comfort of familiarity, a concept known as the exposure effect (24). A student peer coach’s lack of deep familiarity with current practice allows innovation to occur without the exposure effect (24). Additionally, fear of retaliation is a common reason employees avoid voicing their opinions (25), but external student peer coaches can focus on the objective of improving workflow and implementing a new program without this concern.

Implementation leadership, such as a site champion, often results in successful implementation of a new program; these champions are typically employees of the institution (26). To promote sustainability of new clinical services, peer coaches can aid in identifying and training a site champion to be responsible for the program over the long-term. In addition to providing benefits to the pharmacy, this student peer coaching intervention gave students valuable experience in leadership and project development. The students in this intervention were paid interns, but the coaching process can be extrapolated to any student in project-based work. Those students can serve either as an implementation site champion or a peer coach.

The most striking result of this study lies in the observed improvement in productivity and efficiency. The total number of hours per week spent on the MTS program by coached pharmacy personnel declined despite an increase in the total number of personnel involved in the MTS program and an increase in overall MTS program engagement. After excluding the outlier pharmacy, the average number of completed patient visits and revenue per pharmacy were significantly higher in the coached group than in the uncoached group. Regardless of the outlier, the average percentage of total patient encounters that resulted in a completed visit was significantly higher in coached versus uncoached pharmacy teams. This finding suggests that pharmacy personnel were better engaged when student peer coaches supported implementation. Spending less time while achieving better outcomes is a result of successful integration of the MTS program into pharmacy workflow.

Interest is growing in the role of student pharmacists in implementation of patient care programs in community pharmacies. A study in Pennsylvania examined the effect of a student-run quality engagement team in supporting initial implementation of comprehensive medication management programs in community pharmacies. The study found that biweekly calls with the student-run quality engagement team increased the number of patient encounters completed by pharmacy personnel during the implementation period (27). This finding suggests that student pharmacists can be a key asset in program implementation, a conclusion further supported by the results of our study.

Although this study showed peer coaching as a promising approach to implementation, it has several limitations. First, we did not collect demographic information from survey respondents, which means we could assess differences in responses based only on pharmacy site characteristics, not respondent characteristics. Second, pharmacy leaders opted into the student peer coaching intervention, which may have introduced bias in the comparison of coached and uncoached pharmacy teams. Pharmacists who agreed to participate in the coaching intervention may have been more willing at baseline than nonparticipants to implement the MTS program into workflow. Third, because pharmacies with the largest number of potential patient encounters were initially targeted for coaching, it is possible the larger number of available opportunities may have affected willingness to engage with the MTS program overall. Fourth, we did not include formal power calculations in our evaluation because of the small number of pharmacies that qualified for inclusion. Fifth, pharmacies differed substantially in patient volume, patient population, available personnel, and other clinical services offered; although program flexibility allowed coaches to address specific needs, this flexibility reduced consistency in the approaches used by student coaches. A significant outlier in engagement data highlights how pharmacy-specific characteristics can affect overall involvement in the MTS program. The reasons for the success of the outlier pharmacy are being explored to identify best practices that can be implemented both with and without the assistance of peer coaches.

To better understand the effect of peer coaching, future studies could collect subjective data (perceived success, readiness for change, and attitudes) from an uncoached control group. Additional studies could explore the effect of peer coaching on patient outcomes, such as HbA_1c_ or blood pressure. Insight into these areas would highlight where peer coaching best fits in with the implementation of novel clinical services in community pharmacies. Studies with large samples and a randomized enrollment process might reduce the risk of unintended bias.

With increased rates of chronic disease and reduced availability of health care providers, finding alternative ways to offer primary care is key to improving health outcomes. Healthy People 2030 identifies high-priority objectives, known as Leading Health Indicators (LHIs), to improve health and well-being in the US (28). Interventions designed around these LHIs can substantially improve health outcomes. Reducing the number of yearly diabetes diagnoses and increasing blood pressure control in adults are 2 LHIs (28). The LHI measure for blood pressure control is still in the preliminary data collection stage; the number of yearly diabetes diagnoses has increased in the past 5 years (29,30). Chronic disease is especially prominent in rural areas, which typically have less access to health care providers; the prevalence of diabetes ranges from 9% to 17% higher in rural areas than in urban areas (31). Hypertension rates in rural areas also exceed the rates in urban areas, with rates being highest in the Southeast (32).

The accessibility offered by community pharmacies provides a way to connect patients to care, but improved outcomes cannot occur without effective implementation. Student pharmacists serving as peer coaches allow for pharmacist-led clinical services to be integrated into traditional pharmacy workflows, creating an environment in which community pharmacies can help fill a gap in the management of chronic conditions.
